# Biology and bionomics of malaria vectors in India: existing information and what more needs to be known for strategizing elimination of malaria

**DOI:** 10.1186/s12936-019-3011-8

**Published:** 2019-12-03

**Authors:** Sarala K. Subbarao, Nutan Nanda, Manju Rahi, Kamaraju Raghavendra

**Affiliations:** 1 Indian Council of Medical Research (ICMR), Ramalingaswami Bhavan, New Delhi, India; 2 ICMR-National Institute of Malaria Research (NIMR), Sector-8, Dwarka, Delhi India; 3 Present Address: Delhi, India

**Keywords:** *Anopheles*, Malaria vectors, Sibling species, Species complexes, Vector control, Malaria elimination, Ecosystems, Vector bionomics, Early biting, Outdoor transmission

## Abstract

India has committed to eliminate malaria by 2030. The national framework for malaria elimination released by the Government of India plans to achieve this goal through strategic planning in a phased manner. Since vector control is a major component of disease management and vector elimination, it requires a thorough understanding of the biology and bionomics of malaria vectors exhibiting definite distribution patterns in diverse ecosystems in the country. Although a wealth of information is available on these aspects, lesser-known data are on biting time and rhythm, and the magnitude of outdoor transmission by the vectors which are crucial for effective implementation of the key vector control interventions. Most of the data available for the vector species are at sensu lato level, while the major vectors are species complexes and their members distinctly differ in biological characters. Furthermore, the persistent use of insecticides in indoor residual spray and long-lasting insecticidal nets has resulted in widespread resistance in vectors and changes in their behaviour. In this document, challenges in vector control in the Indian context have been identified and possible solutions to overcome the problem are suggested. Adequate addressing of the issues raised would greatly help make a deep dent in malaria transmission and consequently result in disease elimination within the targeted time frame.

## Background

As per the World Malaria Report 2018, in 2017 80% of the global malaria burden was borne by 15 countries in sub-Saharan Africa and India. India (with 4% malaria cases) is one of the five countries that contributed 50% malaria cases worldwide. The other four countries are Nigeria (25%), Democratic Republic of the Congo (11%), Mozambique (5%), and Uganda (4%) [1]. Total malaria cases reported in 2017 in India were more than 9.5 million and this number is 3 million cases fewer than those reported in 2016. *Plasmodium falciparum* cases were 63.38% and the other major parasite species was *Plasmodium vivax* (about 33–34%); *Plasmodium malariae* and *Plasmodium ovale* continued to be about 2–3% of the total cases. *Plasmodium knowlesi* cases are so far reported only from Andaman and Nicobar Islands.

## National framework for malaria elimination

In line with global developments in achieving the elimination of malaria in different countries, India has committed to eliminating malaria by 2030. The National Framework for Elimination of Malaria in India [2] was released on 16 February, 2016. Based on the epidemiological data of 2014, 15 states and Union territories (UTs) with annual parasite incidence (API) less than 1 are placed under Category 1, 11 with API less than 1 and 1 or more districts with API more than 1 under Category 2, and 10 with API more than 1 under Category 3. The national malaria control programme plans to achieve elimination in a phased and strategic manner, and prevent re-establishment of local transmission of malaria in areas where it has been eliminated and sustain a malaria-free status nationally by 2030. In order to reach the timely milestones and ultimately the elimination by 2030, key interventions recommended for the three categories are listed in detail in the document, and the vector control forms the major component of malaria control.

With the use of indoor residual spray (IRS) and long-lasting insecticidal nets (LLINs) in association with prompt case detection and treatment facilities, there has been a reduction in the incidence of malaria cases in the country. Epidemiological data of the country shows that certain areas still report intense malaria transmission [1].

Of the different technical and operational reasons identified for failure of control, lack of relevant knowledge on the behaviour of vectors could be one. There are reports from several countries [3–6] and from India [7, 8] showing drift in mosquito behaviour to rest outdoors owing to the use of insecticide interventions, especially LLINs. The concern is that the effectiveness of these tools would eventually be compromised. Reports on change in the behaviour of vectors are a warning and require attention to achieve elimination target. Hence, outdoor transmission and residual malaria are receiving renewed focus globally.

In the context of India’s commitment to malaria elimination by the year 2030, there is a need to revisit the existing knowledge on biology and bionomics of malaria vectors. The recently published monograph *Guidelines for malaria vector control* [9] states that “accurate species identification is crucial for all studies and surveillance activities on field populations of vectors. Many of the vectors belong to species complexes and require advanced molecular analyses for species identification, necessitating appropriate laboratory resources. Without accurate species identification, data collected on behaviour, distribution and infection rates for decision-making by control programmes will have limited use”.

Extensive studies have been carried out in different parts of the country and several books, articles, and reviews have been published (to mention a few: [10–13]). The objective of writing this review is to describe the biological characters and bionomics of major malaria vectors to highlight the changes that have occurred in the species prevalence and biological characters, as this information is important for planning vector control strategies. In this article, studies that need to be carried out to generate data to fill the gaps in existing knowledge in vector biology and bionomics, and generate data to quantify behavioural aspects to facilitate informed decisions in selecting tools/strategies to interrupt transmission effectively are suggested. Furthermore, mechanisms to integrate existing tools, additional vector control interventions to complement the existing ones, with a focus to address biological aspects of vectors are discussed in this review article.

## Vector species prevalence in India

Six *Anopheles* species, *Anopheles baimaii*, *Anopheles culicifacies*, *Anopheles fluviatilis*, *Anopheles minimus*, *Anopheles stephensi*, and *Anopheles sundaicus* are implicated as primary vectors transmitting malaria in different eco-geographical regions of India. In addition, the secondary/local vectors *Anopheles annularis*, *Anopheles nivipes*, *Anopheles philippinensis*, and *Anopheles varuna* transmit malaria along with either one or two major vectors in different parts of the country. Anopheline vector fauna of this country is further enriched by the recognition of certain of these vector species as species complexes [11, 13]. The anopheline species that have been found as complexes and the members in each of these complexes found in India are: Culicifacies Complex (A, B, C, D, E), Dirus Complex (two-*An. baimaii* in the northeast and *Anopheles elegans* in the south), Fluviatilis Complex (S, T, U, V), Minimus Complex (*An. minimus*), Sundaicus Complex (species D), Annularis Complex (A and B) and Subpictus Complex (A, B, C, D) [11, 13]. These species, owing to their distinct biological characters and ecological preferences, show a specific distribution pattern. Malaria epidemiology in India is complex and the endemicity varies distinctly in diverse ecosystems of the country. The ecosystems vary in the proportions of two predominant malaria parasites *P. falciparum* and *P. vivax*, and the prevalence of the six major *Anopheles* vector species and their sibling species along with one or two vectors of local importance.

## Eco-geographical distribution of major malaria vectors

The major ecosystems where malaria is endemic in the country are forests, rural plains, urban, coastal, and arid areas. There is a strong relationship between ecosystem and vector and parasite species prevalence to the malaria transmission in an area. Vector species have a distinct distribution pattern in the country and the pattern is governed by land use patterns and type of breeding sites available. For example, topography and climatic conditions in the forest eco-systems, in addition to influencing the prevalence of vector species, also affects longevity of the vectors. In laboratory studies at 27–28 °C and 70–80% relative humidity, *P. falciparum* takes longer to complete its sporogonic cycle than *P. vivax* in vectors [14] and there were similar observations in laboratory feeding experiments done at NIMR (unpublished). As all malaria vector species in India transmit both the major parasite species *P. falciparum* and *P. vivax* the differences observed in the prevalence of parasite species are due to variable climatic conditions in the ecosystems prevalent in the country.

Broadly, ecosystems and the major malaria vectors and their sibling species observed in different states are given in Table 1.

**Table 1:** Major malaria vector species prevalent in different ecosystems in India

Ecosystem	Major vector species and sibling species observed in ecosystems	Regions/States
Rural plains, undulating plains	*An. culicifacies* A, B, C, D, E (sibling species with variable prevalence exhibit specific sympatric associations)	Entire country
Plain and undulating forests (deep valleys, hills and hillocks with thick forests)	*An. culicifacies* B, C, D+* An. fluviatilis* S, T	Central and eastern regions: Madhya Pradesh, Chhattisgarh, Jharkhand
Hilly-forested terrains	*An. fluviatilis* S, T + *An. culicifacies B,* C, E *An. minimus *+ *An. fluviatilis* S, T	Eastern region: Odisha, Chhattisgarh and Andhra Pradesh Eastern region—parts of Odisha
Forest and forest-fringe areas of northeast	*An. baimaii* *An. baimaii* + *An*. *minimus*	All northeastern states Northeastern states: Arunachal Pradesh, Assam, Manipur, Meghalaya, Mizoram, Nagaland and Tripura
Foothill regions	*An*. *minimus*	Northeastern states
Deforested areas where rice cultivation is prevalent	*An. minimus *+* An. culicifacies* s. l.	Northeastern states: Assam, Manipur, Meghalaya, Sikkim
Peri-urban areas	*An. stephensi *+* An. culicifacies* s. l.	Delhi, Goa, Tamil Nadu, etc.
Urban and semi-urban areas	*An. stephensi*—3 ecological forms—type form, intermediate form, var. *mysorensis*	Andhra Pradesh, Delhi, Goa Maharashtra, Kerala, Telangana, Tamil Nadu, West Bengal
Arid zone	*An. stephensi*—type form and var. *mysorensis*	Rajasthan, Gujarat
Island ecosystem areas with brackish water and freshwater breeding places	*An. sundaicus* species D (cytotype D)	Andaman and Nicobar Islands

## Biology and bionomics of major malaria vector species and their sibling species in different ecosystems

*Anopheles culicifacies* and *An. fluviatilis* are major vectors contributing to 75–80% malaria in India, and *An. culicifacies* alone is responsible for 60–70% of malaria. In a hilly-forested ecosystem, *An. fluviatilis* is the major vector species with *An. culicifacies* in the secondary role, and in plain and forest-fringe areas *An. culicifacies* is the predominant vector species. In certain plain areas, *An. culicifacies* is the only species transmitting malaria. Biology and bionomics studies carried out in India broadly reveal that *An. culicifacies* and *An. fluviatilis* predominantly rest indoors and bite indoors [10, 15–17]. *Anopheles culicifacies* is observed in high densities (per man-hour densities) and is predominantly a zoophilic species, and indoor-resting collections from cattle sheds are generally more than those from human dwellings [10, 17, 18]. In contrast, densities of *An. fluviatilis* are low with significantly higher numbers in human dwellings than in cattle sheds. This species has a high human blood index (HBI) ranging between 0.8 and 0.9 in areas where it has been implicated in malaria transmission [17, 19–22]. In Balaghat district of Madhya Pradesh, *An. culicifacies* tested positive for *Plasmodium* antigen, almost in equal number from cattle sheds and human dwellings. Furthermore, from outdoor light traps and indoor light traps positives were found. Of the total 67 *An. fluviatilis* collected, 3/13 positives were found from human dwelling pyrethrum spray collections [23]. In district Sundergarh (Odisha), in the forested areas, human biting rates (HBR) of *An. culicifacies* and *An. fluviatilis* were 0.6 and 6.47 per person per night, respectively, and in plain areas where only *An. culicifacies* was found, its HBR was the same as in forest areas [21]. In areas where both *An. culicifacies* and *An. fluviatilis* transmit malaria, as in hilly, forested villages of Odisha, low densities of *An. fluviatilis* from April to September are compensated by *An. culicifacies*, and from October to February–March *An. fluviatilis*, with its highest densities of the year, transmit malaria leading to perennial transmission [17, 21, 24].

These studies establish the distinct difference in intensity of malaria transmission in plain and forest areas in the same district, and also show distinct difference in transmission efficiencies of these two vector species. Similar observations were made in Madhya Pradesh in areas of Mandla and Dindori districts, which are different in terrain and forest cover [25]. *Anopheles culicifacies* was incriminated from both the districts, while only *An. fluviatilis* from evergreen forests of Dindori. In Madhya Pradesh, in whole night collections using light traps, *An. culicifacies* and *An. fluviatilis* were collected in outdoor traps [23, 25]. There was no difference in the number of *An. culicifacies* in indoor and outdoor light traps, while a higher number of *An. fluviatilis* was collected in outdoor traps than in indoor resting collections, suggesting the preferential exophilic nature of this species. In Panna district (Madhya Pradesh), families that spent about 3 weeks in the forest for the collection of mahua flowers (*Madhuca indica* used for making liquor) returned with falciparum malaria infection [26]. These studies suggest the occurrence of outdoor transmission of malaria in forest areas of central India. In Assam, Meghalaya, Manipur and Sikkim States in the northeast where deforestation was done for agricultural purpose and rice cultivation is in practice, *An. culicifacies* was found with higher sporozoite rates than *An. minimus.* Irrigation channels for rice cultivation were one of the important breeding sites for *An. culicifacies*, and seen to be responsible for the increased presence of this vector in these States [27]. Similarly, in Thar Desert area of northwestern Rajasthan, with the development of canal-irrigation system, *An. culicifacies* established itself as a vector [28]. However, *An. stephensi* continues to be the major vector in irrigated and non-irrigated villages in these areas of Rajasthan [29].

Of the five sibling species identified in the Culicifacies Complex, except species B (which is either a poor or non-vector), all other species (A, C, D, E) transmit malaria in different parts of the country [23, 30–34]. Epidemiological and laboratory susceptibility studies support the poor vector status of species B [35, 36]. All these sibling species have a distinct distribution pattern in the country with species B prevalent in all the areas surveyed either exclusively or in sympatric association with other sibling species [11, 13]. Host feeding preference studies of species A, B, C, and D showed them to have low HBI ranging between 0 and 0.05 [13, 37]. Distinct seasonal variations in prevalence were observed among the sibling species [11, 13, 18]. Among the four sibling species examined for the biting rhythm, species A and B showed peak biting activity in the second quarter of the night, between 22:00 and 24:00 h, in all the seasons. Species C and D showed a different biting rhythm with peak biting in the first quarter between 18:00 and 21:00 h in April [13, 38]. In Chhattisgarh, Madhya Pradesh and Odisha, species C is the predominant sibling species [17, 21, 31]. In Madhya Pradesh, biting in early hours (first quarter of the night) was 60% for species C and 30 to 40% for the next predominant vector species, species D [38]. For species A, B, C, and D, the proportion of biting in the first quarter was highest in April (biting rhythms of species E has not been studied so far). In Madhya Pradesh, in April, active malaria transmission of *P. vivax* and *P. falciparum* cases was observed where species C was predominant [39], and in Jharkhand, transmission was reported in April, although *An. culicifacies* was in low densities [40].

Among the four members S, T, U, and V of the Fluviatilis Complex, species S was found with very high anthropophagy (90–98%) and positivity to *P. vivax* and *P. falciparum* infections [11, 17, 19]. Species T is predominantly zoophilic [19], and a few specimens positive for *P. vivax* and *P. falciparum* sporozoite antigen were found in forest villages of Madhya Pradesh [23]. Sporozoite antigen-positive specimens of species U have not been found so far. Species V was identified in district Hardwar in Uttarakhand State in sympatric association with species T and U. In the indoor collections, 70% specimens of species V were collected from human and mixed dwellings and its HBI was 0.04, while in the same sample collection, species T and U were totally zoophilic [41]. Because of distinct differences in the distribution of these sibling species and their feeding preference, great variation in the role of *An. fluviatilis* sensu lato in malaria transmission was observed in the country. *Anopheles fluviatilis* species S is a highly efficient sibling species of this complex and the major vector in hilly, forested villages of Chhattisgarh [20] and Odisha [8, 17, 21, 24, 33]. However, in recent studies in Keonjhar and Sundergarh districts in Odisha State in contrast to earlier observations, species T was predominant and along with species S it was found in higher numbers in cattle sheds than in human dwellings [7, 8]. In the Singhbhum hill area in Keonjhar district, *An. minimus* was reported along with *An. fluviatilis* species S (90%) and species T (9.1%) in indoor and outdoor human landing catches for the first time outside of the northeast [42, 43]. *Anopheles minimus* and *An. fluviatilis* S were observed throughout the year and were highly anthropophilic with 92 and 90.2% human blood positivity, respectively. Recently, *An. minimus* has been found in other districts in India, West Singhbhum district, Jharkhand (MK Das, pers. comm.) and Kalahandi district, Odisha (RK Hazara, pers. comm.). *Anopheles annularis* is a secondary vector in Odisha. This taxon is a complex of two sibling species, A and B [44].

*Anopheles baimaii* and *An. minimus* are vectors in the northeastern states. *Anopheles baimaii* is reported from all the states in the northeast (Sikkim, unpublished) [45]. *Anopheles baimaii* which is predominantly exophilic rests during day time on tree trunks/creepers in forests. It bites indoors and outdoors, and it briefly rests indoors on walls for about 20–30 min before biting [46]. Recently it has been collected indoors in large numbers in the State of Tripura [47, 48]. It is highly anthropophilic species and in Assam its HBI ranged from 0.667 to 1.0 during different months (average 0.923) [46]. Biting was observed in all the four quarters of the night with about 6–7% in the first quarter, 75% of biting in the second and third quarters, and 20% in the fourth quarter. In this study in Assam, 21% of overall effective entomological inoculation rate (EIR) was seen in the first quarter of the night [49]. *Anopheles elegans*, the second member in the Dirus Complex reported from southern India, has not been incriminated as vector so far. Presently only one sibling species of the Minimus Complex, *An. minimus* is reported from India. This species is reportedly endophilic, endophagic and highly anthropophagic and is found in low densities ranging from < 1 to 7 mosquitoes per man-hour in indoor resting collections [50]. Preference to bite humans was very high (93%) and sporozoite rate was 3.3% with sporozoite positives found during all months of the year. Biting activity was observed throughout the night with peak biting after midnight, between 01:00 and 04:00 h. In the northeastern states, in addition to *An. baimaii* and *An. minimus*, *An. nivipes* and *An. philippinensis* play a secondary role in the transmission of malaria. *Anopheles nivipes* was observed in Arunachal Pradesh, Assam, Manipur, Meghalaya, and Nagaland [51]. Between these two closely related mosquito species under the Annularis Group, *An. nivipes* was predominant in Assam and Nagaland, while *An. philippinensis* was more prevalent in the states of Mizoram and Arunachal Pradesh. *Anopheles nivipes* was incriminated as a vector of *P. falciparum* in Nagaland bordering Assam, and both these species were reported exophilic in behaviour and predominantly zoophilic [52].

*Anopheles sundaicus* is now found only in Andaman and Nicobar Islands and was not reported from the mainland after its last report in 1974–75 from South 24 Parganas district in West Bengal [10]. Prior to its disappearance from mainland, this species was reported from Andhra Pradesh, Odisha and West Bengal [10]. This is a species complex, and only one species, species D (cytotype D) was found in the islands [53, 54]. It has a low preference to bite humans, with HBI of 0.025, but in exclusive human dwelling collections, HBI was 0.18 [55]. It was collected indoors from both human dwellings and cattle sheds, and also from outdoors. Exophagy and bimodal biting activity with peak biting around 23.00 h and the second peak around 02.00 h were observed [56]. In a recent study conducted in the Andaman and Nicobar Islands, *An. sundaicus* was found positive for *P. knowlesi* [57].

The three forms of *An. stephensi* distinguished on the basis of ridge number on floats of eggs are type form predominant in urban areas, intermediate in semi-urban areas, and var*. mysorensis* in rural areas [58]. As no mating barrier was observed in laboratory crosses between the three forms, and that egg morphological and chromosomal inversion polymorphism studies in rural and urban areas suggested the three forms to be differentially found in different ecosystems, they were referred as ecological forms [58–60]. *Anopheles stephensi* is the major malaria vector in urban areas and transmits at low densities [61]. While this species is the major vector in arid zones of rural Rajasthan [28, 29], it is considered as a poor/non vector in the rural areas of other parts of India [58]. In certain parts of Iran *An. stephensi* var*. mysorensis* was found as the only vector transmitting malaria [62]. In these areas, animal hosts were very low in number or were totally absent. Recently this species is speculated to be a complex based on examination of odorant binding protein 1 intron I sequence in *An. stephensi* specimens collected from Iran and Afghanistan [63]. The three biological species recognized as species A, B and C correspond to type form, intermediate form and var*. mysorensis*, respectively. The main strategy to interrupt malaria transmitted by *An. stephensi* in urban areas by the National Vector Borne Disease Control Programme (NVBDCP) under the urban malaria scheme is larval control, and in rural areas of Rajasthan where this species is reported resting and biting indoors, indoor residual spraying is used. In Rajasthan, in pre-DDT era this species was found resting on the walls of the houses, but recently in Jodhpur district of Rajasthan this species was found resting on household objects (hanging clothes, furniture, stacked clothes, etc.) avoiding walls both in insecticide sprayed and unsprayed villages, suggesting change in its resting behaviour [64]. Furthermore, *An. stephensi* biting was observed outside the houses in courtyards during dusk, and it was found entering the houses after 23.00 h and most of the entry was between 01.00 and 04.00 h. In Goa State, *An. stephensi* is the major vector. In this state *An. stephensi* could not be collected from well-built houses, and large collections were made from huts near construction sites [65]. In human landing catches inside the houses, seasonal variations were observed in biting times [66]. In Chennai city, *An. stephensi* is the vector and this city contributes 60–70% of malaria cases of Tamil Nadu State [67]. In one of the high malaria-endemic areas of Chennai, higher densities of *An. stephensi* were observed in cattle sheds in the vicinity of human dwellings than in human dwellings [68]. Maximum mosquito collections were from houses with thatched roofs and only about 5% were from houses with asbestos and tiled roofs. In addition to *An. stephensi*, *An. subpictus* was found positive for sporozoites in coastal areas of Goa [69] and in Chennai [68]. In Goa, sporozoite positive specimens were identified as species B of *An. subpictus*. *Anopheles subpictus* is reported to be a complex of 4 sibling species [70], and species B, which is a coastal species, was earlier incriminated in Puducherry [71].

## Vector control strategies in use

Under NVBDCP, India vector control has been playing an important role in disease management. The two main vector control strategies that are being used are indoor spraying with residual insecticides (IRS) and LLINs targeting adult mosquitoes in rural areas of the country. In urban areas, where vector breeding is in defined and confined habitats, larval control using chemical insecticides, bacterial pesticides and larvivorous fish is the applied strategy. In the northeastern states and in forested areas of the states in Central India, LLINs are being distributed to saturation.

## Current situation on responses of major vectors to vector control tools

For indoor residual spraying, DDT, malathion and pyrethroids have been introduced in the malaria control programme in a sequential order. In 1959 *An. culicifacies* was reported resistant to DDT [72], to hexachlorocyclohexane [73] and in 1973, to malathion [74]. The differential development of resistance among the sympatric sibling species under similar selection pressure was observed [11, 15, 75–77]. Now that IRS has been in practice for more than six decades, vector species have developed increased levels of resistance to one or more insecticides in a given area depending on the use of insecticides and the selection pressure exerted on the vector species. In a recent review of the resistance/susceptible status of vector species to different insecticides, it is mentioned that *An. culicifacies* in the rural plains have exhibited widespread resistance [78]. In 70% of the districts examined, this species has shown resistance to at least one insecticide, while in some to two and in some other districts to all three classes of insecticides. Resistance to pyrethroids has been found widespread in Chhattisgarh, Madhya Pradesh and Odisha while in other states it was sporadic. With reference to other vector species: *An. fluviatilis* in hilly, forested and foothill areas, where it is the major vector, was mostly susceptible to DDT, in one district even to malathion and fully susceptible to pyrethroid; *An. baimaii* and *An. minimus* (except in one district resistant to DDT), which are major vectors in the northeastern states, were fully susceptible to all the three insecticides; *An. sundaicus* was reported to be resistant to DDT and malathion in Car Nicobar; and, *An. stephensi,* a vector in urban areas, is susceptible to Temephos, used for larval control, and is also susceptible to bacterial pesticide *Bacillus thuringiensis israelensis* (*Bti*) [78]. Scaling up of LLIN intervention has brought about changes in sibling species composition, resting behaviour and feeding preferences of *An. fluviatilis* in Odisha [7, 8].

## Challenges and possible solutions to effective vector control

To achieve effective vector control towards elimination, among various confounding factors it is important to identify the challenges pertaining to the biological and bionomic characters of vectors and related operational issues. Those identified and the few more that require attention are listed in Table 2, in order to facilitate taking informed decisions on strategies to be used to limit transmission.

**Table 2:** Challenges identified and possible solutions for effective vector control

S. No.	Challenges	Recommendations—studies on vector biology and bionomics, choosing appropriate vector control tools from existing ones and identifying newer tools where needed
1	Changes in the ecosystem due to developmental activities, and continuous pressure of insecticides on vector species have led to changes in the vector species prevalence and also in their biological characters	Situational analysis of vector species prevalence and their bionomics especially to generate data on seasonal density of vector species, resting (indoor and outdoor) and biting (endophagy and exophagy) behaviour, biting rhythm, EIRs, insecticide susceptibility: i. Desirable to carry out studies in all the districts which have more than one API in the Categories 2 & 3. Keeping in view the human and financial resource constraints, studies to be taken up in states with high incidence of malaria ii. In these areas *An. culicifacies* and *An. fluviatilis* are major vectors and both are species complexes—(a) identification of sibling species of these complexes and (b) bionomics of vectors species as mentioned above at the sibling species level iii. (a) In the north eastern states bionomics studies of *An. baimaii* and *An. minimus* in selected areas and (b) the relative role of *An. culicifacies* and its sibling species and their responses to LLINs main usage which is now the main control strategy iv. In Andaman & Nicobar Islands detailed vector bionomics studies in the islands endemic for malaria
2	Early biting and seasonal variations in the biting rhythms i. *An. culicifacies* species C and D ii. *An. baimaii*	i. Indoor and outdoor all night biting rhythm studies during different seasons in the states where species C and species D are reported ii. a. In all the states of north east—proportion biting indoors/outdoors during different seasons and during different quarters of the night in order to estimate the outdoor biting and seasonal variations if any ii. b. Quarter-wise EIRs to quantify the magnitude of transmission ii. c. If LLINs are being used and early biting is observed (indoors and outdoors), additionally IRS using different class of insecticide to which the species is susceptible to be done in the houses/cattle sheds to target the early biting mosquitoes which would go for resting indoors ii. d. Promoting use of personal protection repellents during dusk time
3	Outdoor resting and transmission i. Outdoor transmission in jhum cultivation areas—*An*. *baimaii, A*n*. minimus* ii. *An. baimaii*—predominantly exophilic found resting in forests iii. Transmission in forest areas where people go to collect produce	i. Data on vector biting away from houses in forest areas/in jhum cultivation areas and establishing the quantum of transmission outdoors through EIRs ii. Delimiting the areas where *An. baimaii* is the exclusive vector—introduction of vector control tools that can target exophilic mosquitoes iii. a. Identification of vectors in the forest areas of Madhya Prade*s*h and estimation of transmission that is originating from forest areas. Other forest areas in central and eastern region (Chhattisgarh, Andhra Pradesh and Jharkhand states) are to be examined for peoples movement to forest areas and relate with epidemiology iii. b. Selection of tools to target exophilic and forest dwelling vectors
4	Changes in the behavior of vectors As saturation of areas with LLINs distribution is being used as main vector control strategy in high endemic states, and because of the properties of pyrethroids, changes in the vectors with respect to resting and biting times are expected as observed in Odisha	Regular surveillance of vector species in LLINs distributed areas for changes in the species and sibling species composition and in behavior with reference to resting sites, host feeding preference and biting time
5	*An. subpictus* in Goa and Chennai, and also in other coastal areas where malaria transmission is reported	Biology and bionomics study of this species, and assessment of its relative role in transmission in coastal areas
6	*P. knowlesi* transmission dynamics *P. knowlesi* positive blood smears found in different islands of Andaman & Nicobar islands—Port Blair, Car Nicobar, Teresa and Campbell Bay. *An. sundaicus* has been found positive for *P. knowlesi*	i. Assessing the possible role of other vector species in the transmission of *P. knowlesi*. Establishing site of transmission and initiating in depth studies on biology and bionomics of vector species and identification of suitable strategies to control the vector ii. The transmission cycle in the islands—Examination of *Macaca fascicularis* (the primary host for *P. knowlesi*) present on the islands for the presence of parasite, and establishing its transmission mode: zoonotic and/or human to human infections
7	IRS for the control of *An. culicifacies* and *An. fluviatilis* *An. culicifacies* sibling species are predominantly zoophagic and cattle shed collections are high. Furthermore, with the use of LLINs *An. fluviatilis* has shifted its resting to cattle sheds. As per the current strategy for IRS, only human dwellings are sprayed	Where IRS is the choice for the control of vectors, spraying in all the resting places including the cattle sheds
8	Development of resistance to pyrethroids	Primarily development of pyrethroid resistance should be avoided or delayed following WHO norms [78]. To decrease pyrethroids selection pressure, in areas where *An. fluviatilis* and *An. culicifacies* are sympatric and *An. fluviatilis* is susceptible to DDT, instead of LLINs, DDT can be sprayed to control *An. fluviatilis* effectively. To control *An. culicifacies* if is highly resistant to DDT, feasibility of spraying one round of pyrethroid in pre-monsoon months could be studied
9	Operational issues related to IRS Quality and dosage of insecticide, poor coverage, following schedules etc.	i. Training to spray personnel for quality spraying ii. Health education to the community on importance of spray coverage iii. Use of compression sprayers
10	Number of spray cycles and time of spraying As per the NVBDCP guidelines two rounds of spray one in May–June and another in July–August/September	In the areas where *An. fluviatilis* and *An. minimus* are vectors, and their prevalence extends into January and February and malaria cases are noticeably high, extra round of IRS i.e. total three rounds to interrupt the transmission. Spray cycles to be rescheduled to cover the transmission seasons/months
11	Insecticide resistance management With the continuous and increasing use of pyrethroids in indoor sprays and in ITNs/LLINs, resistance to pyrethroids has been observed in the major vector *An. culicifacies*. IRS and LLINs distribution in the same areas is precipitating resistance to pyrethroids. In addition, continuous use of DDT and malathion has resulted in high levels of resistance to these insecticides in *An. culicifacies*, and also to some degree in other vectors	i. Regular surveillance of vector dynamics and resistance monitoring to plan resistance management strategies ii. New insecticide classes are needed to manage resistance to existing insecticides. Avoiding simultaneous use of LLINs and IRS with pyrethroids or same class of any insecticides
12	*i. An. stephensi* in semi-arid rural areas of Rajasthan—exophily, exophagy and resting on house hold objects and avoiding resting on walls of houses ii. At construction sites and at other developmental project execution sites that increase mosquito productivity	i. Bio-environmental methods—environmental management and manipulation, source reduction, larvivorous fish etc.; larviciding (wherever possible) replacing the current strategy of IRS or in addition to IRS ii. IRS or LLINs can be used depending on the type of housing and people’s acceptance. Source reduction and use of larvicides or larvivorous fish in water storing containers
13	Protection to people in areas away from houses from mosquito bites Providing protection to people (i) living in the forests (ii) who live away from their homes for short periods for the collection of forest produce (North-east and Madhya Pradesh) or for Jhum cultivation (north east) and (iii) who get bitten by mosquitoes outside away from the houses (iv) during early hours of morning or in the evening	At present there are no tools available in India to provide protection to those who live in forests and are not accessible to protection with the two main tools that are being used by the programme, IRS or LLINs Following tools that have been tested in other malaria endemic countries could be evaluated for their efficacy and suitability in Indian settings i. Insecticide impregnated tents, plastic tarpaulin, canvas polyethylene tents which could be used in special situations ii. Insecticide impregnated sheets, blankets and personal clothes, hammocks etc. iii. Spatial repellent dispensers iv. Totally mosquito proof portable huts. v. Attractive toxic sugar baits (ATSB) vi. Eave tubes etc.

## Conclusions

The use of indoor spray with residual insecticides and LLINs to target adults is the cornerstone of the national malaria control programme. The efficacy of these interventions depends on the biological characters of the vector species, such as resting and feeding behaviour. The success of IRS depends exclusively on the indoor resting (endophilic) behaviour of vector species irrespective where they feed, while for LLINs it depends on site of use (indoor/outdoor) and on feeding time of biting. Considering that the major vectors are mainly indoor resting and endophagic, these strategies are being implemented against all the vector species in the country. In the light of widespread resistance in *An. culicifacies* to the three classes of insecticides in use for interventions, there is an urgent need to implement novel strategies to overcome resistance [79], which includes use of insecticide molecules with novel modes of action for the management of resistance and for the effective control of vector species. Soon interventions using combinations of synergists with insecticides and mixtures containing new class of insecticides for IRS and LLINs will be available. The data presented in the section on biology and bionomics points out the presence of populations of vector species that are exophilic and early biting. Cultural and agricultural practices in certain endemic areas make people vulnerable to biting by vector species that are exophilic and early biters. To address outdoor and early biting of vector species there is an urgent need for new tools and to evaluate them for their efficacy and feasibility to use in different ecosystems. In recent years in different endemic countries newer tools are being tested for their efficacy, such as spatial dispensers using volatile pyrethroids [80] and for treating eve ribbons and odour-baited traps [81], eave tubes [8], totally mosquito-proof portable huts for the protection of rice cultivators [82], attractive toxic sugar baits [83, 84], etc., are being evaluated in malaria-endemic countries. Insecticide-impregnated sheets, blankets, personal clothes, and hammocks, etc., can be used to protect people who stay in forests for specific occupations. Novel and emerging tools for species-specific control include *Wolbachia*-based disease control strategy, sterile insect technique (SIT), incompatible insect technique (IIT), gene drive technology, etc. While SIT and IIT are used for population suppression, *Wolbachia* transinfected mosquitoes and those that are modified using gene drive/editing technology can be used for population replacement to control the disease they transmit. A strain of *An. stephensi* transinfected with *Wolbachia* from *Aedes albopictus* showed refractoriness to *P. falciparum* [85]. Another strain of *An. stephensi* that was genetically engineered to express genes targeted against the malaria parasite *P. falciparum* using CRISPR–Cas9 system interrupted the development of *P. falciparum* [86]. Both these strains are yet to be field-tested. Species-specific tools, although very effective, have a limitation: in many areas more than one vector transmits malaria and many of the major malaria vectors are species complexes. This necessitates the need for the release of more than one species strain in an area. These techniques are advantageous in areas where only one species is responsible for the transmission, and because they could provide protection from disease while not attempting species elimination.

With intensive control activities to reach the elimination target, regular surveillance of vectors for changes in prevalence of vector species and their behavioural aspects, and regular monitoring of insecticide resistance should be made routine activities by the programme. The need of the hour is to identify the knowledge gap and to generate data to fill it. Equally important is to test new tools for their efficacy and their suitability in different ecosystems the vector species are occupying.

## Data Availability

Not applicable

## Abbreviations

API: annual parasite incidence
ATSB: attractive toxic sugar baits
*Bti*: *Bacillus thuringiensis israelensis*
CRISPR–cas9: clustered regularly interspaced short palindromic repeats–CRISPR associated protein 9
DDT: dichlorodiphenyltrichloroethane
EIR: entomological inoculation rate
HBI: human blood index
HBR: human biting rate
IIT: incompatible insect technique
IRS: indoor residual spraying
LLIN: long-lasting insecticidal net
NVBDCP: National Vector Borne Disease Control Programme
SIT: sterile insect technique
UT: union territory
WHO: World Health Organization

## References

[1] WHO. World malaria report 2018. Geneva: World Health Organization; 2018. https://www.who.int/malaria/publications/world-malaria-report-2018/en/. Accessed 23 Nov 2018.

[2] NVBDCP. National framework for malaria elimination in India (2016–2030). Directorate of National Vector Borne DIseases Control programme (NVBDCP), Directorate General of Health Services (DGHS), Ministry of Health & Family Welfare, Government of India. Accessed 27 July 2017.

[3] Bugoro H, Hii JL, Butafa C, Iro’ofa C, Apairamo A, Cooper RD (2014). The bionomics of the malaria vector *Anopheles farauti* in Northern Guadalcanal, Solomon Islands: issues for successful vector control. Malar J..

[4] Russell TL, Govella NJ, Azizi S, Drakeley CJ, Kachur SP, Killeen GF (2011). Increased proportions of outdoor feeding among residual malaria vector populations following increased use of insecticide-treated nets in rural Tanzania. Malar J..

[5] Reddy MR, Overgaard HJ, Abaga S, Reddy VP, Caccone A, Kiszewski AE (2011). Outdoor host seeking behaviour of *Anopheles gambiae* mosquitoes following initiation of malaria vector control on Bioko Island, Equatorial Guinea. Malar J..

[6] Thomsen EK, Koimbu G, Pulford J, Jamea-Maiasa S, Ura Y, Keven JB (2017). Mosquito behavior change after distribution of bednets results in decreased protection against malaria exposure. J Infect Dis.

[7] Rath A, Prusty MR, Das M, Mahapatra N, Tripathy H, Hazra RK (2015). A shift in resting habitat and feeding behavior of *Anopheles fluviatilis* sibling species in the Keonjhar district of Odisha, India. Trans R Soc Trop Med Hyg.

[8] Waite JL, Swain S, Lynch PA, Sharma SK, Haque MA, Montgomery J (2017). Increasing the potential for malaria elimination by targeting zoophilic vectors. Sci Rep..

[9] WHO. Guidelines for malaria vector control 2019; Geneva: World Health Organization.www.who.int/malaria/publications/atoz/9789241550499/en/. Accessed 15 May 2019. 30844152

[10] Rao TR (1984). The Anophelines of India.

[11] Subbarao SK. Anopheline species complexes in South-East Asia region. 1998 WHO Technical Publication SEARO. No. 18.

[12] Dash AP, Adak T, Raghavendra K, Singh OP (2007). The biology and control of malaria vectors in India. Curr Sci.

[13] WHO-SEARO. Anopheline species complexes in South and South-East Asia. SEAR Technical publication No. 57 New Delhi: World Health Organization, Regional Office for South-East Asia; 2007. https://apps.who.int/iris/handle/10665/204779. Accessed 1 Mar 2012.

[14] Patz JA, Olson SH (2006). Malaria risk and temperature: influences from global climate change and local land use practices. Proc Natl Acad Sci USA.

[15] Subbarao SK, Sharma VP (1997). Anopheline species complexes and malaria control. Indian J Med Res.

[16] Singh N, Mishra AK, Chand SK, Sharma VP (1999). Population dynamics of *Anopheles culicifacies* and malaria in the tribal area of central India. J Am Mosq Contr Assoc..

[17] Nanda N, Yadav RS, Subbarao SK, Joshi H, Sharma VP (2000). Studies on *Anopheles fluviatilis* and *Anopheles culicifacies* in relation to malaria in forest and deforested riverine ecosystems in northern Orissa, India. J Am Mosq Contr Assoc..

[18] Subbarao SK, Vasantha K, Adak T, Sharma VP (1987). Seasonal prevalence of sibling species A and B of the taxon *Anopheles culicifacies* in villages around Delhi. Indian J Malariol.

[19] Nanda N, Joshi H, Subbarao SK, Yadav RS, Shukla RP, Dua VK (1996). *Anopheles fluviatilis* complex: host feeding patterns of species S, T and U. J Am Mosq Contr Assoc..

[20] Nanda N, Bhatt RM, Sharma SN, Rana PK, Kar NP, Sharma A (2012). Prevalence and incrimination of *Anopheles fluviatilis* species S (Diptera: Culicidae) in a malaria endemic forest area of Chhattisgarh state, central India. Parasit Vectors..

[21] Sharma SK, Tyagi PK, Padhan K, Upadhyay AK, Haque MA, Nanda N (2006). Epidemiology of malaria transmission in forest and plain ecotype villages in Sundargarh District, Orissa, India. Trans R Soc Trop Med Hyg.

[22] Sahu SS, Gunasekaran K, Vanamail P, Jambulingam P (2011). Seasonal prevalence and resting behaviour of *Anopheles minimus* Theobald & *An. fluviatilis* James (Diptera: Culicidae) in east-central India. Indian J Med Res..

[23] Singh N, Chand SK, Bharti PK, Singh MP, Chand G, Mishra AK (2013). Dynamics of forest malaria transmission in Balaghat district, Madhya Pradesh. India. PLoS ONE..

[24] Sahu SS, Gunasekaran K, Krishnamoorthy N, Vanamail P, Mathivanan A, Manonmani A (2017). Bionomics of *Anopheles fluviatilis* and *Anopheles culicifacies* (Diptera: Culicidae) in relation to malaria transmission in east-Central India. J Med Entomol.

[25] Chand G, Chaudhary NK, Soan V, Kaushal LS, Sharma RK, Singh N (2015). Transmission dynamics and epidemiology of malaria in two tribal districts in Madhya Pradesh, India. Indian J Med Res..

[26] Singh N, Chand SK, Mishra AK, Nagpal AC (2014). Migration malaria associated with forest economy in central India. Curr Sci.

[27] Akhtar N, Nagpal BN, Kapoor N, Srivastava A, Gupta HP, Saxena R (2016). Impact of ecological and climatic changes on vectors of malaria in four North-Eastern states of India. Indian J Ecol.

[28] Tyagi BK (2004). A review of the emergence of *Plasmodium falciparum*-dominated malaria in irrigated areas of the Thar Desert, India. Acta Trop..

[29] Joshi V, Sharma RC, Singhi M, Singh H, Sharma K, Sharma Y, Adha S (2005). Entomological studies on malaria in irrigated and non-irrigated areas of Thar desert, Rajasthan, India. J Vector Borne Dis..

[30] Subbarao SK, Adak T, Vasantha K, Joshi H, Raghavendra K, Cochrane AH (1988). Susceptibility of *Anopheles culicifacies* species A and B to *Plasmodium vivax* and *Plasmodium falciparum* as determined by immunoradiometric assay. Trans R Soc Trop Med Hyg.

[31] Subbarao SK, Vasantha K, Joshi H, Raghavendra K, Usha Devi C, Sathyanarayan TS (1992). Role of *Anopheles culicifacies* sibling species in malaria transmission in Madhya Pradesh state, India. Trans R Soc Trop Med Hyg.

[32] Kar I, Subbarao SK, Eapen A, Ravindran J, Satyanarayana TS, Raghavendra K (1999). Evidence for a new malaria vector species, species E within the *An. culicifacies* complex (Diptera: Culicidae). J Med Entomol..

[33] Tripathy A, Samanta L, Das S, Parida SK, Marai N, Hazra RK, Kar SK, Mahapatra N (2010). Distribution of sibling species of *Anopheles culicifacies s.l.* and *Anopheles fluviatilis s.l.* and their vectorial capacity in eight different malaria endemic districts of Orissa, India. Mem Inst Oswaldo Cruz..

[34] Das M, Das B, Patra AP, Tripathy HK, Mohapatra N, Kar SK (2013). *Anopheles culicifacies* sibling species in Odisha, eastern India: first appearance of *Anopheles culicifacies* E and its vectorial role in malaria transmission. Trop Med Int Health..

[35] Subbarao SK, Vasantha K, Raghavendra K, Sharma VP, Sharma GK (1988). *Anopheles culicifacies*: sibling species composition and its relationship to malaria incidence. J Am Mosq Contr Assoc..

[36] Adak T, Kaur S, Singh OP (1999). Comparative susceptibility of different members of the *Anopheles culicifacies* complex to *Plasmodium vivax*. Trans R Soc Trop Med Hyg.

[37] Joshi H, Vasantha K, Subbarao SK, Sharma VP (1988). Host feeding patterns of *Anopheles culicifacies* species A and B. J Am Mosq Contr Assoc..

[38] Satyanarayan TS. Field and laboratory studies on selected ecological and behavioral aspects of the sibling species of the *An. culicifacies* complex. Ph.D. Thesis. 1996; Dept of Zoology, Delhi University, Delhi.

[39] Singh N, Mishra AK, Shukla MM, Chand SK (2003). Forest malaria in Chhindwara, Madhya Pradesh, central India: a case study in a tribal community. Am J Trop Med Hyg.

[40] Das MK, Prajapati BK, Tiendrebeogo RW, Ranjan K, Adu B, Srivastava A (2017). Malaria epidemiology in an area of stable transmission in tribal population of Jharkhand, India. Malar J..

[41] Nanda N, Singh OP, Dua VK, Pandey AC, Nagpal BN, Adak T, Dash AP (2013). Population cytogenetic and molecular evidence for existence of a new species in *Anopheles fluviatilis* complex (Diptera: Culicidae). Infect Genet Evol..

[42] Jambulingam P, Sahu SS, Manonmani A (2005). Reappearance of *Anopheles minimus* in Singhbum hills of east-central India. Acta Trop.

[43] Sahu SS, Gunasekaran K, Jambulingam P (2009). Bionomics of *Anopheles minimus* and *An. fluviatilis* (Diptera: Culicidae) in east-central India, endemic for falciparum malaria: human landing rates, host feeding, and parity. J Med Entomol..

[44] Atrie B, Subbarao SK, Pillai MKK, Rao SRV, Sharma VP (1999). Population cytogenetic evidence for sibling species in *Anopheles annularis* (Diptera: Culicidae). Ann Entomol Soc Am.

[45] Prakash A, Sarma DK, Bhattacharyya DR, Mohapatra PK, Bhattacharjee KD, Mahanta J (2010). Spatial distribution and r-DNA second internal transcribed spacer characterization of *Anopheles dirus* (Diptera: Culicidae) complex species in north-east India. Acta Trop.

[46] Prakash A, Bhattacharyya DR, Mohapatra PK, Mahanta J (1997). Indoor biting behavior of *Anopheles dirus* Peyton and Harrison 1979 in upper Assam. Mosq Borne Dis Bull..

[47] Dev V, Adak T, Singh OP, Nanda N, Baidya BK (2015). Malaria transmission in Tripura: disease distribution and determinants. Indian J Med Res.

[48] Sarmah NP, Bhowmik IP, Sarma DK, Sharma CK, Medhi GK, Mahapatra PK (2019). Role of *Anopheles baimai*: potential vector of epidemic outbreak in Tripura, North-east India. J Glob Health Rep..

[49] Prakash A, Bhattacharyya DR, Mohapatra PK, Mahanta J (2005). Malaria transmission risk by the mosquito *Anopheles baimaii* (formerly known as *An. dirus* species D) at different hours of the night in North-east India. Med Vet Entomol..

[50] Dev V (1996). *Anopheles minimus*: its bionomics and role in malaria transmission of malaria in Assam, India. Bull World Health Org..

[51] Subbarao SK, Vasantha K, Nanda N, Nagpal BN, Dev V, Sharma VP (2000). Cytotaxonomic evidence for the presence of *Anopheles nivipes* in India. J Am Mosq Contr Assoc..

[52] Bhattacharyya DR, Prakash A, Sarma NP, Mohapatra PK, Singh S, Sarma DK (2010). *Anopheles nivipes* (Diptera: Culicidae) in the transmission of *Plasmodium falciparum* in north–eastern India. Ann Trop Med Parasitol..

[53] Nanda N, Das MK, Wattal S, Adak T, Subbarao SK (2004). Cytogenetic characterization of *Anopheles sundaicus* (Diptera: Culicidae) population from Car Nicobar Island, India. Ann Entomol Soc Am..

[54] Alam MT, Das MK, Ansari MA, Sharma YD (2006). Molecular identification of *Anopheles (Cellia) sundaicus* from the Andaman and Nicobar Islands of India. Acta Trop.

[55] Kumari R, Joshi H, Giri A, Sharma VP (1993). Feeding preferences of *Anopheles sundaicus* in Car Nicobar Island. Indian J Malariol.

[56] Kumari R, Sharma VP (1994). Resting and biting habits of *Anopheles sundaicus* in Car Nicobar Island. Indian J Malariol.

[57] Vidhya PT, Sunish IP, Maile A, Zahid AK (2019). *Anopheles sundaicus* mosquitoes as vector for *Plasmodium knowlesi*, Andaman and Nicobar Islands, India. Emerg Infect Dis..

[58] Subbarao SK, Vasantha K, Adak T, Sharma VP, Curtis CF (1987). Egg-float ridge number in *Anopheles stephensi*: ecological variation and genetic analysis. Med Vet Entomol.

[59] Mahmood F, Sakai RK (1984). Inversion polymorphisms in natural populations of *Anopheles stephensi*. Can J Genet Cytol.

[60] Subbarao SK (1996). Genetics of malaria vectors.

[61] Sharma SN, Subbarao SK, Choudhury DS, Pandey KC (1993). Role of *An. culicifacies* and *An. stephensi* in malaria transmission in urban Delhi. Ind J Malariol.

[62] Oshaghi MA, Yaaghoobi F, Vatandoost H, Abaei MR (2006). *Anopheles stephensi* biological forms: geographical distribution and malaria transmission in malarious region of Iran. Pak J Biol Sci.

[63] Firooziyan S, Djadid ND, Gholizadeh S (2018). Speculation on the possibility for introducing *Anopheles stephensi* as a species complex: preliminary evidence based on odorant binding protein 1 intron I sequence. Malar J..

[64] Nagpal BN, Srivastava A, Dash AP (2012). Resting behaviour of *Anopheles stephensi* type form to assess its amenability to control malaria through indoor residual spray. J Vector Borne Dis..

[65] Sumodan PK, Kumar A (2004). Yadav RP Resting behavior and malaria vector incrimination of *Anopheles stephensi* in Goa, India. J Am Mosq Contr Assoc..

[66] Korgaonkar N, Kumar A, Yadav RS, Kabadi D, Dash AP (2012). Mosquito biting activity on humans and detection of *Plasmodium falciparum* infection in *Anopheles* *stephensi* in Goa, India. Indian J Med Res..

[67] Kumar DS, Andimuthu R, Rajan R, Venkatesan MS (2014). Spatial trend, environmental and socioeconomic factors associated with malaria prevalence in Chennai. Malar J..

[68] Thomas S, Ravishankaran S, Justin JA, Aswin A, Mathai MT, Valecha N (2017). Resting and feeding preferences of *Anopheles stephensi* in an urban setting, perennial for malaria. Malar J..

[69] Kumar A, Hosmani R, Jadhav S, de Sousa T, Mohanty A, Naik M (2016). *Anopheles subpictus* carry human malaria parasites in an urban area of Western India and may facilitate perennial malar transmission. Malar J..

[70] Suguna SG, Rathinam KG, Rajavel AR, Dhanda V (1994). Morphological and chromosomal descriptions of new species in the *Anopheles subpictus* complex. Med Vet Entomol.

[71] Panicker KN, Bai MG, Rao USB, Viswam K, Murthy US (1981). *Anopheles subpictus*, vector of malaria in coastal villages of South-East India. Curr Sci.

[72] Rahman J, Roy ML, Singh K (1959). Development of increased tolerance to DDT in *Anopheles culicifacies* Giles, in the Panch Mahal district of Bombay state (India). Indian J Malariol.

[73] Patel TB, Rao TR, Halgeri AV, Deobhankar RB (1958). A preliminary note on a probable case of dieldrin resistance in *Anopheles culicifacies* in Thana district, Bombay state. Indian J Malariol..

[74] Rajagopal R (1977). Malathion resistance in *Anopheles culicifacies* in Gujarat. Indian J Med Res.

[75] Subbarao SK, Vasantha K, Sharma VP (1988). Responses of *Anopheles culicifacies* sibling species A and B to DDT and HCH in India: implications in malaria control. Med Vet Entomol.

[76] Raghavendra K, Vasantha K, Subbarao SK, Pillai MK, Sharma VP (1991). Resistance in *Anopheles culicifacies* sibling species B and C to malathion in Andhra Pradesh and Gujarat States, India. J Am Mosq Contr Assoc..

[77] Raghavendra K, Subbarao SK, Vasantha K, Pillai MK, Sharma VP (1992). Differential selection of malathion resistance in *Anopheles culicifacies* A and B (Diptera: Culicidae) in Haryana State, India. J Med Entomol..

[78] Raghavendra K, Poonam SV, Vaishali V, Elamathi N, Barik TK, Bhatt RM (2017). Temporo-spatial distribution of insecticide-resistance in Indian malaria vectors in the last quarter-century: need for regular resistance monitoring and management. J Vector Borne Dis..

[79] WHO. Global plan for insecticide resistance management in malaria vectors. Geneva: World Health Organization; 2012. https://www.who.int/malaria/publications/atoz/gpirm/en/. Accessed 15 May 2019.

[80] Ogoma SB, Mmando AS, Swai JK, Horstmann S, Malone D, Killeen GF (2017). A low technology emanator treated with the volatile pyrethroid transfluthrin confers long term protection against outdoor biting vectors of lymphatic filariasis, arboviruses and malaria. PLoS Negl Trop Dis..

[81] Mmbando AS, Batista EPA, Kilalangongono M, Marceline F, Finda MF, Mwang EP (2018). Evaluation of a push–pull system consisting of transfluthrin-treated eave ribbons and odour-baited traps for control of indoor and outdoor-biting malaria vectors. Malar J..

[82] Swai JK, Finda MF, Madumla EP, Lingamba GF, Moshi IR, Rafiq MY (2016). Studies on mosquito biting risk among migratory rice farmers in rural south-eastern Tanzania and development of a portable mosquito-proof hut. Malar J..

[83] Müller GC, Schlein Y (2008). Efficacy of toxic sugar baits against adult cistern-dwelling *Anopheles claviger*. Trans R Soc Trop Med Hyg.

[84] Stewart ZP, Oxborough RM, Tungu PK, Kirby MJ, Rowland MW, Irish SR (2013). Indoor application of attractive toxic sugar bait (ATSB) in combination with mosquito nets for control of pyrethroid-resistant mosquitoes. PLoS ONE..

[85] Bian G, Joshi D, Dong Y, Lu P, Zhou G, Pan X (2013). *Wolbachia* invades *Anopheles stephensi* populations and induces refractoriness to *Plasmodium* infection. Science.

[86] Gantz VM, Jasinskiene N, Tatarenkov O, Fazekas A, Macias VM, Bier E (2015). Highly efficient Cas9-mediated gene drive for population modification of the malaria vector mosquito *Anopheles stephensi*. Proc Natl Acad Sci USA.

